# Deposition of zinc oxide as an electron transport layer in planar perovskite solar cells by spray and SILAR methods comparable with spin coating†

**DOI:** 10.1039/c9ra01839e

**Published:** 2019-07-04

**Authors:** M. Dehghan, A. Behjat

**Affiliations:** Photonics Research Group, Engineering Research Centre, Yazd University Yazd Iran abehjat@yazd.ac.ir; Atomic and Molecular Group, Faculty of Physics, Yazd University Yazd Iran

## Abstract

CH_3_NH_3_PbI_3_ planar-structure perovskite solar cells were fabricated with the configuration FTO/ZnO/CH_3_NH_3_PbI_3_/Au. ZnO nanoparticles were synthesized by the precipitation method. Three different deposition methods including spin-coating, spraying and successive ionic layer adsorption and reaction (SILAR) were applied to fabricate the ZnO films as electron transport layers. Certain analyses, such as XRD, SEM, FESEM, UV-visible and *I*–*V* measurements, were carried out to evaluate the performance of the cells. The best cell performance was achieved for the perovskite solar cell with a ZnO film coated by the spin method. The average efficiency was 7% without using any hole transport materials and 10.25% using spiro-OMeTAD as a hole transport material. The average efficiencies of the cells coated by the spraying and SILAR methods using spiro-OMeTAD, were found to be 8.64% and 7.7% respectively. This study demonstrates the versatility of the spray and SILAR coating methods and their potential for fabricating low-cost, large scale, flexible and mass produced perovskite solar cells.

## Abstract 2

We have examined the versatility of spray and SILAR coating methods in fabricating mass produced low cost efficient planar perovskite solar cells.

## Graphical abstract

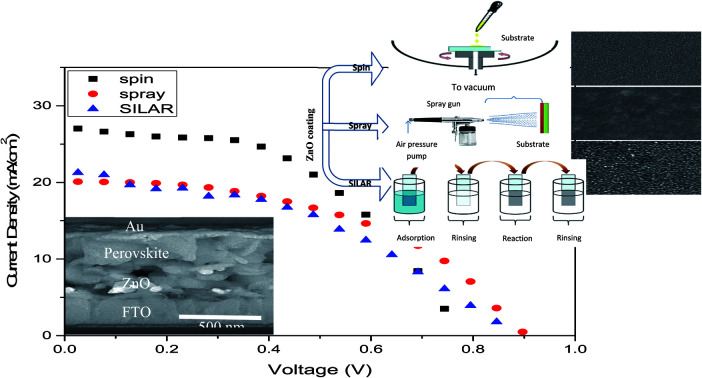

## 1. Introduction

During recent years, perovskite solar cells (PSCs) employing methylammonium lead halide have been considered as promising light harvesters to be used in the field of next-generation photovoltaics. This is due to their competitive efficiency and low-cost manufacture. In 2009, Miyasaka^1^ used organic–inorganic lead halide perovskite compounds as visible-light sensitizers in mesoporous structures and achieved an initial power conversion efficiency (PCE) of 4%. Later, other researchers reached very high efficiencies in a very short time, up to a recent certified record of 22.1%.^2–4^ A perovskite material has very attractive optical and electrical properties which makes it suitable for photovoltaic applications. These properties include long diffusion length up to 175 μm, high carrier mobility, direct optical band gap, broad absorption range, low-cost processing, and ease of fabrication.^5–7^ Different deposition techniques, such as one-step,^8,9^ two-step sequential^10–12^ and vapor deposition methods,^13–15^ have been developed in order to produce high-quality surfaces for perovskite active layers. Vacuum evaporation is considered as a good technique, but it is not advantageous for low-cost solar cell fabrication. On the other hand, one-step deposition of perovskite layers, as compared to the other methods, makes the device fabrication easier and less expensive.^16^

In order to fabricate perovskite solar cells, different types of electron transport layers (ETLs) and hole transport layers (HTLs) have been employed recently. The ETL commonly used in perovskite-based solar cells is titanium dioxide (TiO_2_).^17–19^ TiO_2_ films require sintering at 500 °C before use and this temperature is too high for fabrication of the perovskite solar cells on flexible substrates.^15,20^ This problem is one of the barriers for commercialization of perovskite solar cells. Zinc oxide (ZnO) can be a suitable alternative for TiO_2_ as an electron transport material in PSCs.^21^ ZnO is a semiconductor with a wide band gap and electron mobility higher than that of TiO_2_, which makes it a promising candidate for electron transport layers.^22^ ZnO nanoparticle films can be deposited easily by spin coating,^23,24^ and they need no sintering step. This makes ZnO suitable for coating on flexible substrates and mass production.^19^ The spin coating method produces smooth and high-quality films but is not suitable for large-scale production. Spray and SILAR techniques are other methods of coating which are advantageous in that they are able to produce large-scale cells. SILAR technique is economical and highly feasible for large-area deposition. Also, wastage of chemicals can be avoided.^25–27^

In this research, ZnO nanoparticles were synthesized by a simple precipitation method in the Yazd photonics research lab. Three different coating methods including spin coating, spraying and SILAR method are employed for deposition of ZnO nanoparticle layers to be used as ETLs in perovskite solar cells. To have a reasonable surface of ZnO in spraying method a set-up with speed and distance adjusting options was designed. Particular attention has been paid to producing low-temperature planar perovskite solar cells with ZnO layers coated by spraying and SILAR methods, which are comparable in efficiency with the common method, namely spin coating. According to our research, the cells performances for SILAR and spray methods do not show very big differences with spin coating method. It is desirable to optimize coating methods and make a suitable choice of electron transport material (ETM), which is low-cost, easy to fabricate and usable for large scale and flexible substrates.

## 2. Experimental details

### 2.1. Synthesis of ZnO

First, ZnO nanoparticles (NPs) powder was synthesized through a low-cost and simple precipitation process. A single-step process with large-scale production without unwanted impurities is desirable for the cost-effective preparation of ZnO NPs. ZnO nanoparticles were prepared using zinc acetate (Aldrich) and KOH (Aldrich) as precursors (synthesis of ZnO; ESI†).

### 2.2. Cell fabrication

The Fluorine-doped tin oxide (FTO) coated glass substrates with the sheet resistance of 15 Ω sq^−1^ were patterned by etching with zinc powder and HCl (2 M). The etched substrates were each cleaned under sonication in a diluted detergent, acetone, ethanol and isopropanol for 15 minutes and rinsed with deionized water.^28^ Then, they were dried at 500 °C for an hour. In this study, three different methods including spin-coating, spraying and SILAR method were used for the ZnO deposition. In spin-coating, a ZnO solution was prepared using *n*-butanol, methanol and chloroform as solvents. The solution was then deposited on the substrates by spin-coating at the speed of 3000 rpm for 30 seconds and sintered at 100 °C for 10 minutes. This process was repeated three times to obtain a reasonable layer of ZnO.

To coat ZnO by the spraying method, the ZnO solution was sprayed on the substrates by the manually designed setup (Fig. S1†) with a gentle slope and at a monotonous speed for three times, and each deposited layer was sintered at 100 °C.

In SILAR method, a zinc-ammine solution was prepared for deposition. To prepare it, an ammonia solution (NH_4_(OH) 25%) was added slowly to a 0.1 M ZnSO_4_ solution. This initially forms Zn (OH)_2_ precipitate, but, in excess ammonia, it changes to tetraamine zinc complex [Zn(NH_3_)_4_]^2+^ (eqn (S1)†). The substrates were immersed in the zinc-ammine solution at room temperature and then withdrawn and immersed in deionized water at 90 °C. This cycle was repeated for 15, 20 and 25 times to have a fully covered thin layer of ZnO. The coated substrates were then dried at room temperature.

The deposited ZnO films were used as the electron-transporting layers in cells. The other layers were prepared by spin coating. The structure used here was planar with a FTO/ZnO/CH_3_NH_3_PbI_3_/Au configuration. After ZnO was coated as a hole-blocking layer, the perovskite layer was deposited by the one-step coating method. To prepare the perovskite solution, PbI_2_ powder was solved in a mixture of DMF and DMSO solution at 70 °C. Then, MAI (methylammonium iodide) powder was added, and the resulting solution was spin-coated on top of the ZnO blocking layer. There was no hole transport material used in this process. At last, to finish the cell preparation process and complete the cell structure, 60 nm of gold was thermally evaporated in an ultrahigh vacuum chamber with 10^−6^ mbar on the top of the perovskite layer.

### 2.3. Cell characterization

The crystal structure of the ZnO NPs and their size were determined by X-ray diffraction (XRD) PANalytical X'Pert Pro MPD multipurpose instrument. SEM and FESEM images of the ZnO NPs were taken by a scanning electron microscope (TESCAN, Vega3, Czech) to prove their morphology and crystallization. Absorption spectra of the ZnO films were prepared by an Ocean Optics spectrometer model HR 4000 in order to observe their absorption and calculate the band gap of them. The whole cell was characterized by SEM images and the UV-visible spectra of the perovskite films. The current–density potential (*J*–*V*) of the curves was measured by Keithley Model 2400 under AM 1.5G 100 mW cm^−2^ using a solar simulator from Sharif Solar Co calibrated with a silicon reference cell.

## 3. Results and discussion

**Fig. 1:**
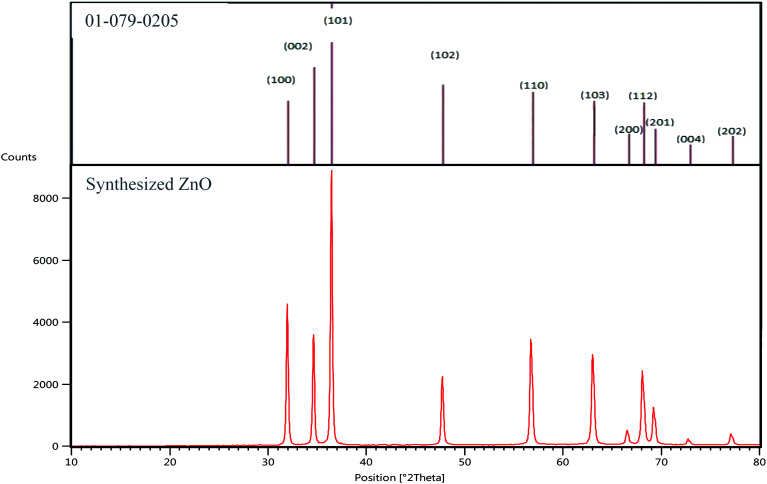
XRD patterns of the synthesized ZnO nanoparticles.

Fig. 1 shows the XRD patterns of the synthesized ZnO nanoparticles. The XRD pattern of the predestined ZnO was used as a reference. Compared with JCPDS cards, the diffraction peaks labeled as (100), (002), (101), (102), (110), (103), (200), (112), (201), (004) and (202) fitted well with the hexagonal ZnO wurtzite structure (reference code: 01-079-0205). As it can be seen in the figure, the peaks of the synthesized ZnO are completely matched with those of the reference. However, the characteristic peaks related to impurities are not detected in the XRD pattern, which confirms the formation of ZnO with high purity.^29–31^

Table 1 shows the microstructural properties for the strongest peaks appearing in the X-ray diffraction pattern of the synthesized ZnO (100, 002 and 101), applied as ETM in this research. The crystallite size was calculated using the Scherer formula^32,33^ as follows:
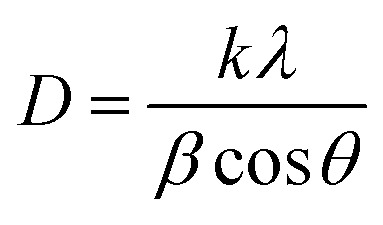
where *D*, *θ*, and *λ* are the mean size of the crystalline domains, the Bragg angle and the incident X-ray wavelength (0.17889 nm) respectively. *K* is a dimensionless shape factor, with a value close to unity (0.9) and *β* is the full-width at half maximum (FWHM) of the diffraction peak.

**Table 1:** The microstructural properties of the synthesized ZnO used as ETM

*hkl*	Position [2*θ*°]	FWHM°	Size [nm]
100	31.96	0.22	37
002	34.62	0.19	42
101	36.43	0.20	41

The average size calculated for the ZnO nanoparticles was 40 nm, which is an appropriate size for the materials used as compact layers in solar cells.

The morphology of the ZnO structure was analyzed using SEM images (Fig. S2†).

**Fig. 2:**
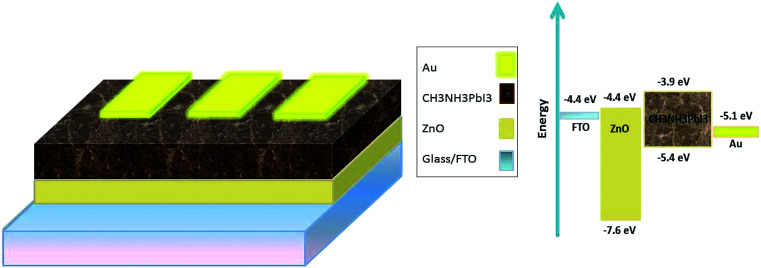
(a) Schematic illustration of the architecture of the perovskite devices fabricated in this study: FTO front contact, ZnO ETL, CH_3_NH_3_PbI_3_ film and Au back contact, and (b) energy levels of the individual device components and possible electronics.^34^

Fig. 2a shows the device structure of our perovskite solar cells grown directly on FTO substrates. A compact layer of ZnO, as an electron transport layer (ETL), was coated with different methods as explained before. The perovskite films were directly coated on the ZnO ETL by a one-step spin-coating method. Following the deposition of the perovskite layer, the cells were finished with thermally evaporated Au back contacts. Fig. 2b demonstrates the energy levels of the layers in the fabricated perovskite solar cells. It can be inferred from the energy diagram that application of ZnO as an ETL matches the perovskite energy level. This facilitates the electron transfer from the absorber layer, which is perovskite here. Moreover, because of its wide band gap, ZnO is a suitable hole-blocking layer matching the perovskite layer. The −7.6 eV valence band in ZnO is also much higher than the −5.4 eV valence band in the perovskite material, resulting in less carrier recombination.

**Fig. 3:**
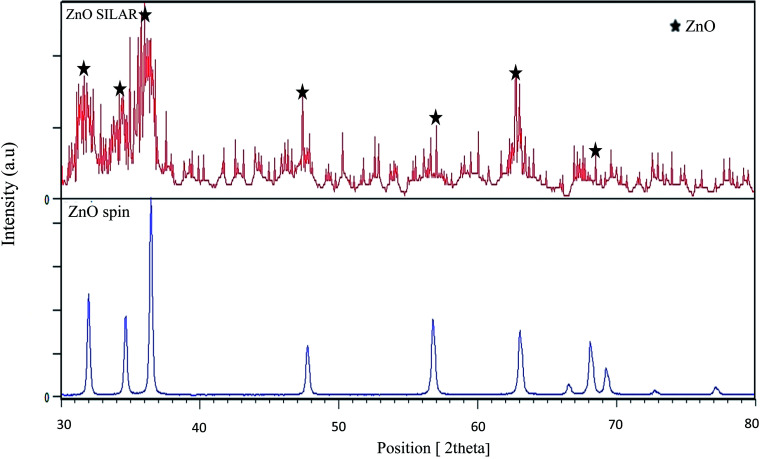
XRD patterns of the ZnO layer deposited by SILAR method compared with spin coating method.

In order to evaluate the crystallinity of the ZnO layer coated by SILAR method, the XRD pattern was provided and shown in Fig. 3. The pattern was provided by grazing incidence X-ray diffraction. As it can be seen from the pattern the ZnO layer deposited by SILAR seems less crystalline compared with ZnO layer deposited by spin.

**Fig. 4:**
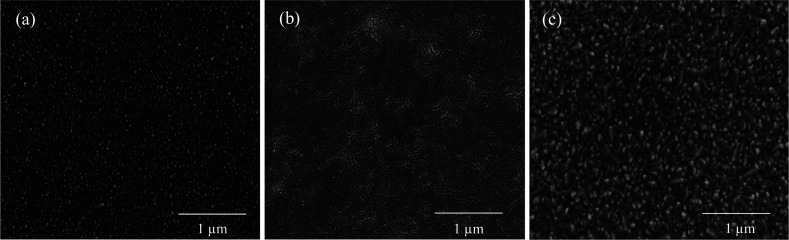
Scanning electron micrographs of the ZnO films deposited on FTO substrates by different coating methods: (a) spin coating, (b) spraying, and (c) SILAR method.

The dependence of the crystallinity, the size of the crystallites and the uniformity of the ZnO thin films on the deposition method was revealed by the SEM micrographs as in Fig. 4. As the figure shows, the surface morphology of the films depends on the method of deposition. Fig. 4a exhibits the microstructure of the films consisting of many spherical grains uniformly distributed throughout the surface with a relatively dense surface structure. Indeed, the surface morphology of the ZnO film deposited by spin coating shows a high density of small grain sizes, which is ideal for application as a compact layer in PSCs. This behavior confirms a similar observation already made.^35–38^ In Fig. 4b, the grains and the grain boundaries have become larger. The layers deposited by spraying exhibit larger and less dense features but very flat and gentle surfaces.^39–41^ The ZnO films coated by the SILAR method (Fig. 4c) have a less uniform and smooth surface than the other two films coated by spin and spray, and the size of the crystals has become larger.^42^ The shape and size distribution of the nanocrystals in each thin film appears to be relatively regular as compared to that in previous reports.^43^

**Fig. 5:**
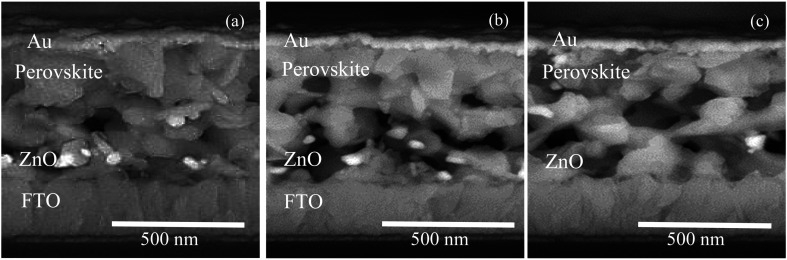
Cross-sectional FESEM images of the fabricated PSCs by different ZnO coating methods: (a) spin coating, (b) spraying, and (c) SILAR method.

The morphology of the perovskite layers of the cells also varied by changing the deposition methods. Fig. 5 shows that the perovskite crystal size with a spin-coated ZnO substrate (Fig. 5a) was almost smaller than the crystal size compared with the cells with the spray-coated (Fig. 5b) and SILAR-deposited (Fig. 5c) ZnO substrate and the perovskite crystals were grown better on the ZnO layer in spin coating method. This can be explained by the ZnO substrate related morphology. The spin-coated ZnO surface also had more uniformity and less porosity as it can be seen in Fig. 5a.

**Fig. 6:**
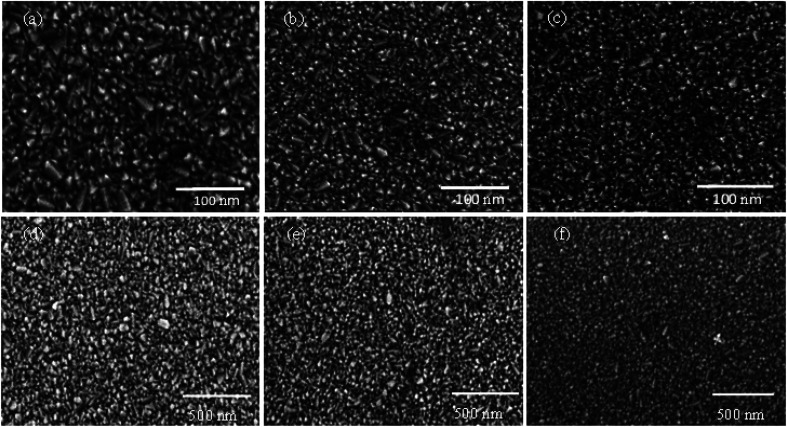
SEM images of the ZnO films deposited on FTO substrates by the SILAR and spray methods. (a)–(c) The SILAR method with 15, 20 and 25 cycles on the scale of 100 nm. (d)–(f) The spray method by 3, 5 and 7 cycles of spraying on the scale of 500 nm.

The SEM images of the ZnO films surfaces coated by the SILAR and the spray methods are presented in Fig. 6. As expected, with an increase in the cycles in the SILAR method, the ZnO surface became denser, and a desirable and more uniform surface was provided for the ETL. Also, the ZnO films coated by the spray method had more circular shapes and denser surfaces in comparison with the films coated by the SILAR method. The best film quality was achieved by seven cycles of spraying.

**Fig. 7:**
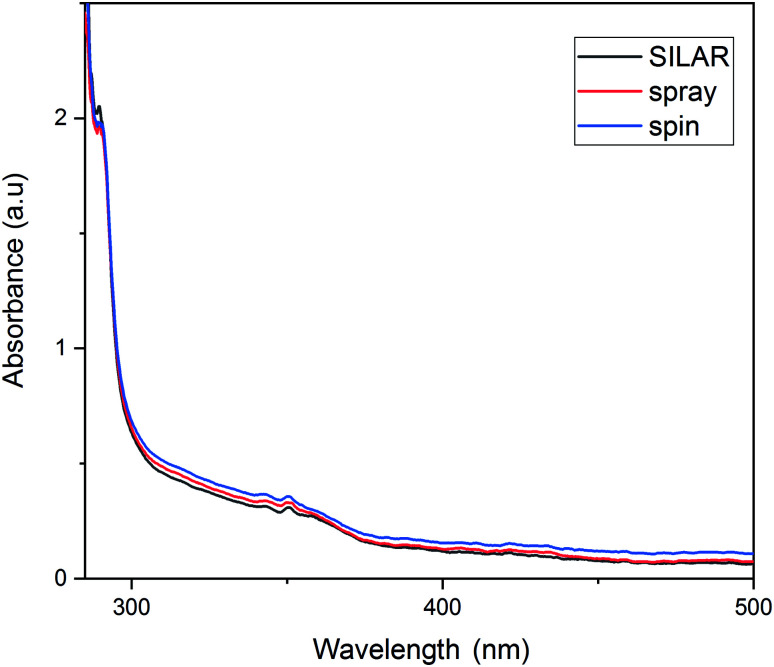
UV-visible spectra of the ZnO thin films coated by different methods.

Fig. 7 plots the absorption spectra of the ZnO thin films based on different deposition methods. The absorption of the films was measured at room temperature in the wavelength range of 300–600 nm. As the graph shows, in all the deposition methods, the films are highly transparent in the visible range, which fulfills the requirements of solar applications well. In all the three methods, the main peak observed is about 350 nm but with a small shift toward intensity. The quasi/total absorption obtained for the wavelengths lower than 370 nm shows a good coverage of the FTO surface. These results are in good agreement with other reports in the literature.^44^ A comparison of the three deposition methods suggested that the ZnO films coated by the spin method had less absorbance and, consequently, more transmittance than the other two methods. According to the graphs and the SEM results, the optical absorbance of the three films would increase with an increase in their crystal size during the growth process. So, it can be concluded that, as the layer quality improves, the transmittance of the layer increases.

**Fig. 8:**
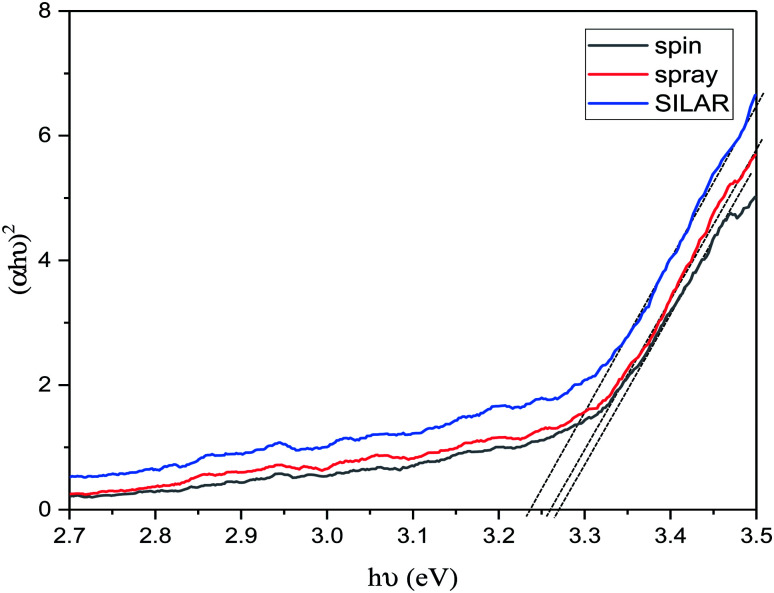
Plot of (*αhν*)^2^*vs.* the photon energy (*hν*) of the ZnO films based on different coating methods. The band gap values are obtained by extrapolating the linear part of the curves.

As presented in Fig. 8, the optical bandgap of the ZnO layers were evaluated, using the Tauc plot (eqn (S2)†). The band gaps were estimated from the intercept of linear portion of the (*αhν*)^2^*vs. hν* plots on *hν* axis. The band gap edge is more and more pronounced as a function of the coating methods. The values serve to validate our crystallite size results according to which a smaller crystallite size is supposed to have a larger bandgap (3.27 eV for the spin-coated ZnO films) and a larger crystallite size is expected to have a smaller bandgap (3.26 eV for the sprayed ZnO layers and 3.24 eV for the ZnO films coated by the SILAR method). On planar surfaces, the Madelung potential is weakened, leading to a reduction of the ionic gap. Its total band gap for ZnO (here 3.24 eV to 3.27 eV), in turn, becomes smaller as compared to the bulk, which is 3.37 eV. This matches well with the commonly agreed value for reference materials.^45,46^

**Fig. 9:**
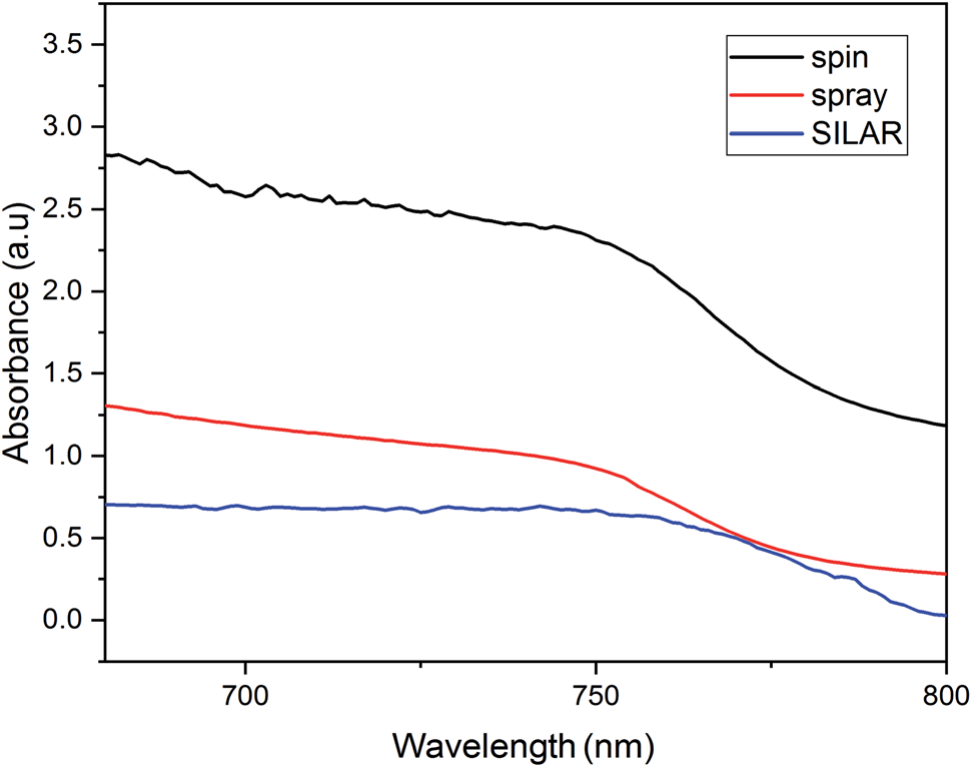
UV-visible spectra of the perovskite layers coated on the ZnO films with different coating methods.

Fig. 9 demonstrates the absorption spectra of FTO/ZnO/CH_3_NH_3_PbI_3_ layer for a series of cells fabricated. What is found from the UV-visible curve verifies the results previously obtained for the ZnO films as a perovskite prelayer. The image shows higher absorbance for the perovskite layer with a spin-coated ZnO substrate. This can be explained by the morphology of the perovskite layer for the spin-coated ZnO substrate (Fig. 5a). The perovskite layer on the top of the spin-coated layer can, thus, absorb much more photons than the perovskite layers of other cells. The SEM images of the spin-coated ZnO layers (Fig. 4) showed a better surface quality in terms of size distribution and smoothness, which helped to have a better growth of the perovskite layer on the ZnO substrate.

**Fig. 10:**
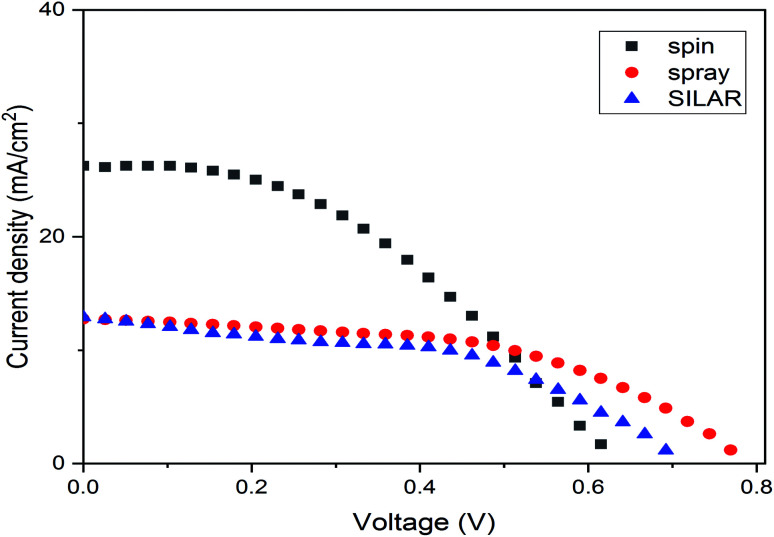
Current density–voltage (*J*–*V*) curves obtained for a FTO/ZnO/CH_3_NH_3_PbI_3_/Au stack with different ZnO deposition methods.

The current density–voltage curves of a series of PCs fabricated with different coating methods for ZnO as a compact layer are presented in Fig. 10, and the detailed photovoltaic parameters such as short-circuit photocurrent (*J*_sc_), open-circuit voltage (*V*_oc_), fill factor (FF) and photon conversion efficiency (PCE) are summarized in Table 2. As it can be seen, the photovoltaic performance of the devices depends on the properties of the ZnO film prepared by different coating methods. The best performance in this research was obtained for the cell with a spin-coated ZnO layer. The *V*_oc_ in the spin method was 0.63. For the SILAR and the spray methods, the *V*_oc_ increased to 0.7 and 0.78 V respectively. The increase in *V*_oc_ was probably due to the lower recombination rate and the larger surface area at the interface. On the other hand, a larger reduction was observed in the *J*_sc_ values through the spray and the SILAR methods in comparison with the spin coating method. It probably hindered the perovskite infiltration into the ZnO film, which eventually resulted in a lower loading of the perovskite. In addition, the films with particles and cracks of irregular shapes on their surface hindered the efficient electron transport, hence showing a lower PCE. All the photovoltaic data were completely in agreement with the SEM and UV spectrum results.^47,48^ Remarkably, the cells based on spin-coated ZnO films demonstrated the highest PCE of 7%, which is much better than that (5.11% and 4.4%) of the cells based on the ZnO thin films deposited by spray and SILAR.

**Table 2:** Average device performance of PSCs with ZnO layers as ETLs deposited by different methods. The averages were obtained by testing five devices

ZnO coating method	*V*_oc_ (V)	*J*_sc_ (mA cm^−2^)	FF (%)	PCE (%)
Spin	0.63	26.25	0.42	0.32 ± 7.00
Spray	0.78	12.74	0.52	0.29 ± 5.11
SILAR	0.70	12.91	0.48	0.41 ± 4.40

**Fig. 11:**
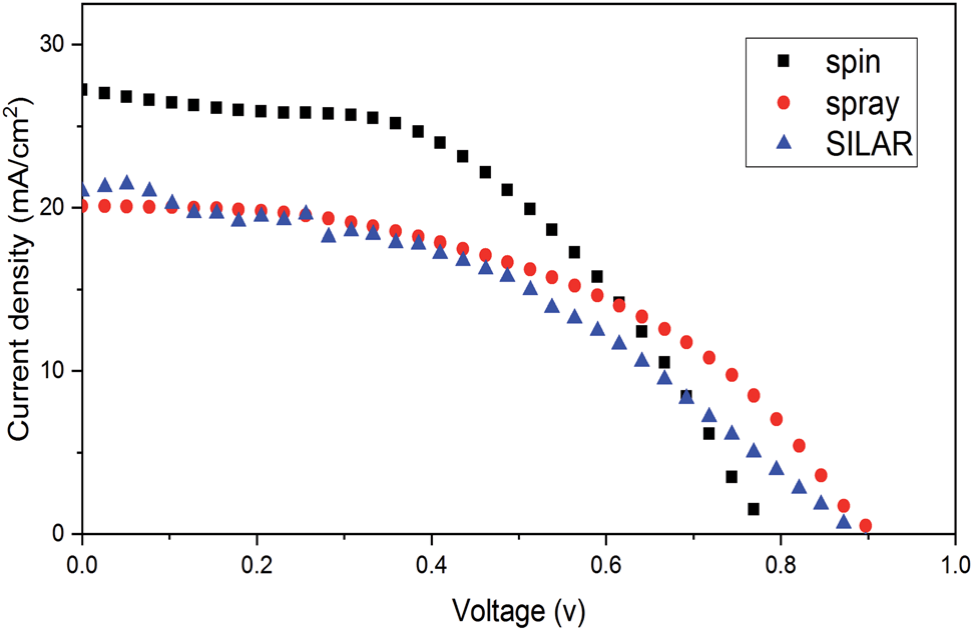
(*J*–*V*) curve obtained for FTO/ZnO/CH_3_NH_3_PbI_3_/spiro-OMeTAD/Au cells with different ZnO deposition methods.

A series of cells were fabricated by using spiro-OMeTAD as a hole transport material to evaluate best performances of all cells. The (*J*–*V*) curve of these series with modified structures is presented in Fig. 11. As it can be seen the top efficiency obtained was 10.25% for spin coating method. The other photovoltaic parameters for champion device including current density, voltage and fill factor for the cells with HTM were found to be 27.25 mA cm^−2^, 0.79 V and 42% respectively.

The detailed photovoltaic parameters for the PSCs with FTO/ZnO/CH_3_NH_3_PbI_3_/spiro-OMeTAD/Au structure and different deposition methods are summarized in Table 3.

**Table 3:** Average device performance of FTO/ZnO/CH_3_NH_3_PbI_3_/spiro-OMeTAD/Au cells deposited by different methods. The averages were obtained by testing five devices

ZnO coating method	*V*_oc_ (V)	*J*_sc_ (mA cm^−2^)	FF (%)	PCE (%)
Spin	0.79	27.25	0.42	10.25 ± 0.25
Spray	0.90	20.01	0.47	8.64 ± 0.35
SILAR	0.88	21.00	0.41	7.70 ± 0.39

## 4. Conclusion

In summary, perovskite solar cells were fabricated at a processing temperature of less than 120 °C. Three most common deposition methods of coating layers were compared to achieve a low-cost, efficient and capable procedure for the mass production of cells and the selection of flexible substrates, which are the requirements for commercialization of perovskite solar cells. According to the results, the cells fabricated by the spin-coating method showed higher efficiency. The performance of the cells made through spray and SILAR coating methods was also noticeable. On the basis of the results, it can be claimed that fabrication of PSCs by applying ZnO, as an electron transport layer, and through spray and SILAR coating methods ensures such advantages as efficiency, low cost, and ease of production. The procedure used in this study can be of benefit for both selection of flexible substrates and large-scale production of solar cells.

## Conflicts of interest

There are no conflicts to declare.

## Supplementary Material

RA-009-C9RA01839E-s001
