# Supplementary material for: Deposition of zinc oxide as an electron transport layer in planar perovskite solar cells by spray and SILAR methods comparable with spin coating
Source: RSC Adv. 2019 Jul 4;9(36):20917–24. doi: 10.1039/c9ra01839e (PMC9065750; doi:10.1039/c9ra01839e)
Supplement: RA-009-C9RA01839E-s001 [file RA-009-C9RA01839E-s001.pdf]

Electronic Supplementary Material (ESI) for RSC Advances.
This journal is © The Royal Society of Chemistry 2019

Synthesis of ZnO

Zinc acetate dihydrate (0.5 g) was dissolved in 20 ml of methanol under stirring at 60˚C, and then solution of KOH (0.25 g of KOH in 10 ml of methanol) was prepared. KOH solution was slowly added to the zinc acetate solution at 60˚C under vigorous stirring for two hours, which resulted in the formation of a white suspension. The white product was centrifuged and washed three times with absolute methanol. Finally, the obtained product was dried at room temperature.

In SILAR method, an ammonia solution ($NH_4(OH)$ 25%) was added slowly to a 0.1 M $ZnSO_4$ solution. This initially forms $Zn(OH)_2$ precipitate, but, in excess ammonia, it changes to tetraamminezinc complex $[Zn(NH_3)_4]^{2+}$ as follows:

(Eq. S1)

$$[Zn(NH_3)_4]^{2+} + 4H_2O \rightarrow Zn^{2+} + 4OH^-$$

$$Zn^{2+} + 2OH^- \rightarrow Zn(OH)_2$$

$$[Zn^{2+} + 2(O^{2-} + H^+)]^{(solid)} + O_2^{gas} \leftrightarrow [Zn^{2+} + O^{2-}]^{(solid)} + (O^{2-} + 2H^+)^{(gas)} + O_2^{gas} =$$

$$[ZnO]^{(solid)} + H_2O + O_2^{gas}$$

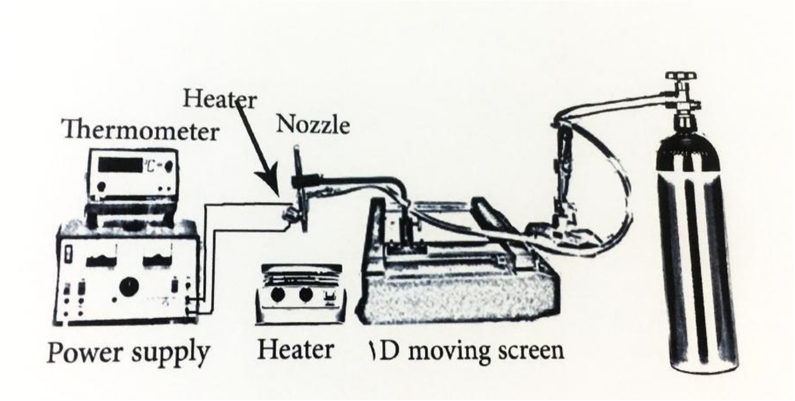

Fig S1. The design for spraying deposition of the ZnO layer

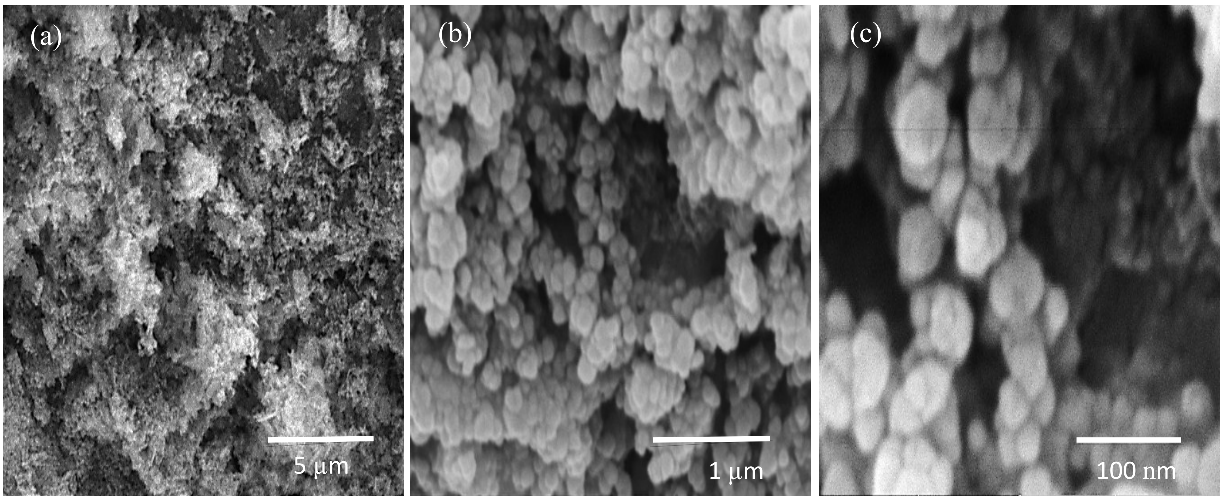

Fig. S2. SEM images of the ZnO synthesized on different magnification scales: (a) 5 μm, (b) 1 μm, and (c) 100 nm.

Tauc equation is given by:

$$(\alpha h\upsilon) = A\,(h\nu - E_g)^n \qquad \text{(Eq. S2)}$$

Where α is the absorption coefficient, $h\upsilon$ is the photon energy, A is the constant, and E_g is the bandgap of the sample. The value of n is ½ or 2 depending upon whether the transition from the valence band to the conduction band is direct or indirect. Here, n is 1/2 because of the direct band gap of ZnO.
